# Supplementary material for: 3’-UTR Polymorphisms of Vitamin B-Related Genes Are Associated with Osteoporosis and Osteoporotic Vertebral Compression Fractures (OVCFs) in Postmenopausal Women
Source: Genes (Basel). 2020 Jun 2;11(6):612. doi: 10.3390/genes11060612 (PMC7349196; doi:10.3390/genes11060612)
Supplement: Supplementary file 1 [file genes-11-00612-s001.pdf]

Supplementary table 1. Stratified effects in the 3'-UTR polymorphisms of *CD320, TCN2, SLC19A1, and SLC19A2* genes on osteoporosis risk

Variables	*CD320* rs9426 CT+TT		*TCN2* rs10418 CT+TT		*SLC19A1* rs1051296 GT+TT		*SLC19A2* rs16862199 CT+TT	
	**AOR (95% CI)[a]**	***P***	**AOR (95% CI)[a]**	***P***	**AOR (95% CI)[a]**	***P***	**AOR (95% CI)[a]**	***P***
Age (years)								
<69	0.815 (0.358-1.860)	0.628	0.764 (0.359-1.627)	0.485	3.059 (1.118-8.373)	0.030	1.182 (0.486-2.877)	0.713
≥69	2.185 (1.021-4.679)	0.044	1.181 (0.574-2.430)	0.652	1.173 (0.364-3.779)	0.789	1.202 (0.578-2.501)	0.622
Hypertension								
No	1.685 (0.832-3.412)	0.147	0.770 (0.360-1.646)	0.500	1.457 (0.505-4.204)	0.486	2.488 (1.119-5.531)	0.025
Yes	0.874 (0.375-2.036)	0.755	1.345 (0.637-2.840)	0.437	1.334 (0.455-3.914)	0.600	0.710 (0.305-1.649)	0.425
Diabetes mellitus								
No	1.330 (0.744-2.376)	0.336	0.892 (0.502-1.585)	0.696	1.766 (0.766-4.073)	0.182	1.655 (0.891-3.076)	0.111
Yes	1.062 (0.273-4.123)	0.931	1.458 (0.348-6.112)	0.606	0.132 (0.009-1.930)	0.139	0.649 (0.179-2.352)	0.511
Vitamin B12[b]								
>395 pg/mL	1.202 (0.610-2.368)	0.596	0.846 (0.418-1.712)	0.642	1.478 (0.725-3.014)	0.283	1.084 (0.526-2.231)	0.827
≤395 pg/mL	1.129 (0.174-7.320)	0.899	0.716 (0.171-2.995)	0.647	0.845 (0.168-4.252)	0.838	2.405 (0.393-14.738)	0.343
Folate[b]								
>4.59 nmol/L	1.379 (0.763-2.490)	0.287	1.143 (0.651-2.008)	0.641	1.620 (0.717-3.658)	0.246	1.569 (0.833-2.955)	0.164
≤4.59 nmol/L	1.399 (0.316-6.198)	0.658	0.718 (0.149-3.463)	0.680	7.418 (0.418-131.780)	0.172	1.476 (0.293-7.442)	0.637
Homocysteine[b]								
<12.68 μmol/L	0.936 (0.513-1.710)	0.830	0.890 (0.503-1.576)	0.690	2.027 (0.901-4.559)	0.088	1.591 (0.857-2.954)	0.142
≥12.68 μmol/L	3.922 (0.970-15.852)	0.055	1.230 (0.319-4.740)	0.763	0.348 (0.033-3.657)	0.379	0.570 (0.153-2.127)	0.403

AOR, adjusted odds ratio; CI, confidence interval.
[a] The odds ratios were adjusted on the basis of risk factors, such as age, hypertension, and diabetes mellitus.
[b] Cut-offs were set at the bottom 15% for vitamin B12 (395 pg/mL) and folate (4.59 nmol/L) and at the top 15% for homocysteine (12.68 umol/L) for both osteoporosis patients and controls.

Supplementary table 2. Stratified effects in the 3'-UTR polymorphisms of *CD320, TCN2, SLC19A1, and SLC19A2* genes on incidence of OVCF

Variables	*CD320* rs9426 CT+TT		*TCN2* rs10418 CT+TT		*SLC19A1* rs1051296 GT+TT		*SLC19A2* rs16862199 CT+TT	
	AOR (95% CI)[a]	*P*	AOR (95% CI)[a]	*P*	AOR (95% CI)[a]	*P*	AOR (95% CI)[a]	*P*
Age (years)								
<69	0.483 (0.181-1.291)	0.147	0.619 (0.252-1.518)	0.295	3.662 (1.187-11.299)	0.024	1.494 (0.594-3.754)	0.394
≥69	3.155 (1.267-7.853)	0.014	1.004 (0.405-2.494)	0.993	0.496 (0.209-1.175)	0.111	1.149 (0.470-2.807)	0.761
Hypertension								
No	1.790 (0.719-4.459)	0.211	0.613 (0.218-1.723)	0.353	0.988 (0.378-2.582)	0.981	2.921 (1.059-8.056)	0.038
Yes	1.055 (0.407-2.735)	0.913	0.981 (0.405-2.378)	0.967	0.959 (0.394-2.331)	0.926	1.016 (0.407-2.536)	0.973
Diabetes mellitus								
No	1.363 (0.671-2.770)	0.392	0.684 (0.331-1.414)	0.306	1.090 (0.531-2.236)	0.815	2.126 (1.023-4.419)	0.043
Yes	1.261 (0.233-6.816)	0.788	2.268 (0.385-13.350)	0.365	0.586 (0.119-2.903)	0.513	0.417 (0.069-2.501)	0.338
Vitamin B12[b]								
>395 pg/mL	0.596 (0.125-2.854)	0.518	0.931 (0.239-3.621)	0.918	1.243 (0.320-4.831)	0.753	0.736 (0.152-3.569)	0.704
≤395 pg/mL	2.052 (0.117-35.864)	0.623	0.415 (0.031-5.649)	0.509	N/A	0.998	6.615 (0.401-109.162)	0.187
Folate[b]								
>4.59 nmol/L	1.097 (0.520-2.315)	0.808	0.984 (0.482-2.010)	0.965	1.028 (0.498-2.120)	0.942	1.575 (0.740-3.351)	0.239
≤4.59 nmol/L	2.040 (0.500-8.322)	0.320	0.413 (0.082-2.073)	0.283	1.291 (0.296-5.625)	0.734	0.562 (0.134-2.360)	0.431
Homocysteine[b]								
<12.68 μmol/L	0.922 (0.443-1.921)	0.829	0.707 (0.343-1.461)	0.349	1.109 (0.549-2.240)	0.774	2.026 (0.993-4.136)	0.052
≥12.68 μmol/L	3.500 (0.795-15.400)	0.097	1.350 (0.285-6.405)	0.706	0.518 (0.090-2.997)	0.463	0.217 (0.037-1.280)	0.092

AOR, adjusted odds ratio; CI, confidence interval.

[a] Odds ratios were adjusted on the basis of risk factors, such as age, hypertension, and diabetes mellitus.

[b] Cut-offs were set at the bottom 15% for vitamin B12 (395 pg/mL) and folate (4.59 nmol/L) and at the top 15% for homocysteine (12.68 umol/L) for both
